# Accumulation of autophagosomes in breast cancer cells induces TRAIL resistance through downregulation of surface expression of death receptors 4 and 5

**DOI:** 10.18632/oncotarget.1174

**Published:** 2013-07-27

**Authors:** Xu Di, Guofeng Zhang, Yaqin Zhang, Kazuyo Takeda, Leslie A. Rivera Rosado, Baolin Zhang

**Affiliations:** ^1^ Division of Therapeutic Proteins, Office of Biotechnology Products, Center for Drug Evaluation and Research, Food and Drug Administration, Bethesda, MD, United States; ^2^ Biomedical Engineering and Physical Science Shared Resource, National Institute of Biomedical Imaging and Bioengineering, National Institutes of Health, Bethesda, MD; ^3^ Confocal Microscopy Core Facility, Center for Biologics Evaluation and Research, Food and Drug Administration, Bethesda, MD

**Keywords:** TRAIL resistance, basal autophagosomes, death receptors, cell surface expression

## Abstract

TNF-related apoptosis-inducing ligand (TRAIL) induces apoptosis through death receptors (DRs) 4 and/or 5 expressed on the surface of target cells. We have previously shown that deficiency of DR4 and DR5 on the surface membrane is a critical mechanism of cancer cell resistance to the recombinant human TRAIL and its receptor agonistic antibodies, which are being evaluated clinically for treating cancers. In certain cancer cells, DR4 and DR5 were found to be mislocalized in intracellular compartments yet to be characterized. Here, we report a novel role of autophagy in the regulation of dynamics of TRAIL death receptors. We first assessed basal levels of autophagosomes in a panel of 11 breast cancer cell lines using complementary approaches (LC3 immunoblotting, RFP-LC3 fluorescence microscopy, and electron microscopy). We found high levels of basal autophagosomes in TRAIL resistant breast cancer cell lines (e.g. BT474 and AU565) and relevant mouse xenograft models under nutrition-rich conditions. Notably, DR4 and DR5 co-localized with LC3-II in the autophagosomes of TRAIL-resistant cells. Disruption of basal autophagosomes successfully restored the surface expression of the death receptors which was accompanied by sensitization of TRAIL-resistant cells to TRAIL induced apoptosis. By contrast, TRAIL-sensitive cell lines (MDA-MB-231) are characterized by high levels of surface DR4/DR5 and an absence of basal autophagosomes. Inhibition of lysosomal activity induced an accumulation of autophagosomes and a decrease in surface DR4 and DR5, and the cells became less sensitive to TRAIL-induced apoptosis. These findings demonstrate a novel role for the basal autophagosomes in the regulation of TRAIL death receptors. Further studies are warranted to explore the possibility of using autophagosome markers such as LC3-II/LC3-I ratios for prediction of tumor resistance to TRAIL related therapies. The results also provide a rationale for future non-clinical and clinical studies testing TRAIL agonists in combination with agents that directly inhibit autophagosome assembly.

## INTRODUCTION

TNF-related apoptosis-inducing ligand (TRAIL) is best known for its ability to induce apoptosis in cancer cells without causing damage to most normal cells [1-3]. This unique property of TRAIL led to multiple clinical trials to evaluate the antitumor potential of recombinant human TRAIL (rhTRAIL) and its receptor-specific agonistic antibodies [4-6]. These therapies act through death receptor (DRs) 4 and/or 5 expressed on the surface of target cells, thereby inducing the assembly of the death inducing signaling complex (DISC) and activation of a caspase cascade. Compared to recombinant human TNFα (rhTNF), which produced severe toxicity after systemic administration [7, 8], TRAIL receptor targeted therapies have demonstrated an improved safety profile in Phase I clinical trials [4-6, 9]. However, some tumor cells (*e.g.* breast cancer) are resistant to TRAIL agonists [10-13]. It is believed that combinational chemotherapies are required to achieve a better clinical efficacy for TRAIL receptor-targeted therapies [14, 15]. Indeed, ongoing phase 2 clinical trials are focused on evaluation of rhTRAIL and DR4 or DR5 monoclonal antibodies in combination with various chemotherapies or targeted therapies [16]. Further concerns arise from the observations that TRAIL treatment even caused an increased growth [17-19] and metastasis [20] of tumor cells that were already resistant to TRAIL induced death. Therefore, it is critical to fully understand the mechanisms underlying TRAIL resistance and to apply the information into the design and selection of combinational drugs to overcome cancer drug resistance towards a better clinical outcome of cancer treatment.

TRAIL resistance can be intrinsic in some tumor cells or acquired in cells that were originally responsive to TRAIL. One of the mechanisms involves tumor characteristics that generally inhibit apoptosis execution such as reduced caspase expression [21, 22], increased expression of caspase inhibitors such as c-FLIP, XIAP, cIAP2 and Bcl-2 [4], and a rapid degradation of truncated Bid (tBid) [23]. Other mechanisms of TRAIL resistance directly related to the defects in the TRAIL receptors themselves, including epigenetic silencing of DR4 [24], dominant-negative mutations in DR4 or DR5 [25], O- and N-linked glycosylation status [26, 27], and co-existence of decoy receptors [28]. Our studies demonstrate that DR4 and DR5 are absent on the cell surface of certain cancer cells despite their total protein expressions [29]. While DR4/DR5 subcellular localizations remain to be characterized, lack of their surface expression appears to be sufficient to render cellular resistance to the corresponding ligands [13, 29]. Additionally, the acquired TRAIL resistance has also been related to deficiency in surface DR4/DR5 resulting, at least partly, from ligand-induced internalization of TRAIL receptors [13, 30] or insufficient receptor trafficking [31] to the cell surface membrane. In line with these observations, several chemotherapy drugs have been shown to enhance TRAIL-induced apoptosis through upregulation of surface expression of DR4 and DR5 in different cancer types [32]. Recent evidence suggests a link between TRAIL resistance and autophagy. Autophagy is a naturally occurring cellular mechanism that degrades aggregated proteins and damaged cellular organelles to maintain cellular homeostasis, while it can also be stimulated in response to pathological and physiological cellular stresses [33]. The sequence of cellular events involves the formation of autophagosomes and fusion with lysosomes to form autolysosomes wherein autophagic cargos are degraded. The process is tightly regulated by a complex signaling network that involves Beclin-1, microtubule-associated protein 1A/1B-light chain 3 (LC3), ATG7, Rab7/9, and other ATG family proteins. It is well documented that tumor cells can activate autophagy in response to cellular stress and/or increased metabolic demands related to rapid cell proliferation [34-37]. Despite its proapoptotic effect in some cases [38, 39], tumor-associated autophagy has been widely implicated in prompting cell growth and chemoresistance [34-36, 40, 41]. This provides a strong basis for clinically testing autophagy inhibitors for cancer treatment [42, 43]. Interestingly, TRAIL has been shown to induce autophagy in different cancer cell lines, including those derived from colon [44, 45], glioma [46], bladder and prostate [47], and breast carcinoma [48, 49]. Furthermore, inhibition of autophagy by pharmacological inhibitors or by silencing *Beclin-1* or *ATG7* genes sensitized TRAIL-resistant cells to TRAIL-induced apoptosis [45, 48].

We envisioned that tumor-associated autophagy might play a role in the regulation of subcellular distribution of TRAIL death receptors. To test this hypothesis, we examined the basal levels of autophagy in a panel of 11 breast cancer cell lines and relevant mouse xenograft models. We found that TRAIL-resistant cells exhibited a high degree of basal autophagy under normal growth conditions. Notably, both DR4 and DR5 were found to co-localize with autophagosomes in TRAIL-resistant cells. Inhibition of autophagosome formation restored the expression of the two death receptors on surface membrane, which was accompanied by a sensitization to TRAIL induced apoptosis. To our knowledge, this is the first evidence that autophagosome formation is directly involved in the downregulation of TRAIL death receptor expression on the cell surface, thereby inhibiting TRAIL-induced apoptosis. These findings shed new light on the role of basal autophagosomes in the regulation of TRAIL apoptosis signaling. The results warrant further studies to determine the potential use of autophagosome markers in prediction of tumor resistance to TRAIL related therapies. Moreover, pharmacological inhibition of basal autophagosome could be evaluated as a novel means for combinational therapies with TRAIL agonists for a better clinical outcome of cancer treatment.

## RESULTS

**Figure 1:**
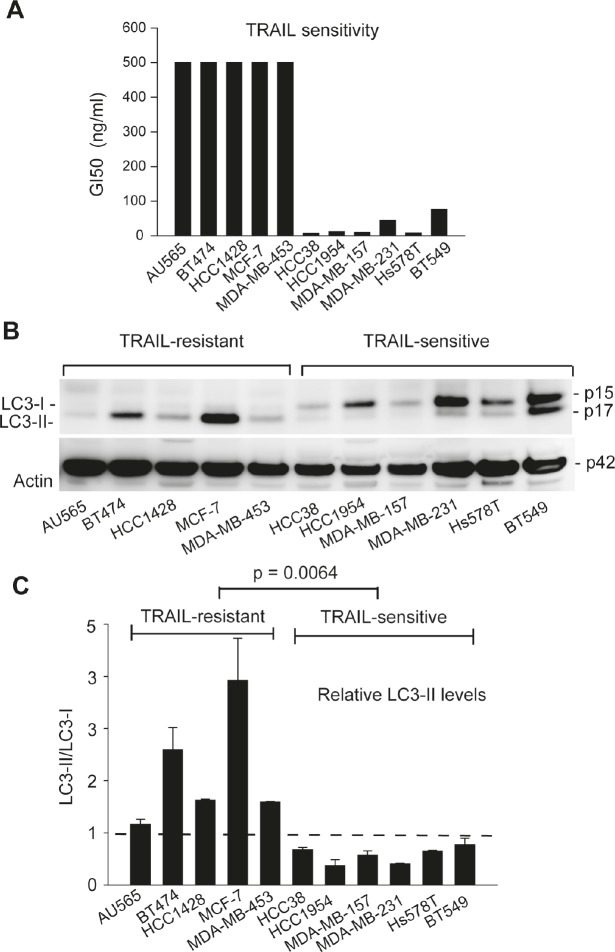
TRAIL resistance correlates with the expression of autophagosome marker LC3-II in breast cancer cell lines (A) Differential sensitivities of the indicated cell lines to TRAIL-induced cytotoxicity. Cells were grown onto 96-well plates and treated with serial doses of rhTRAIL (5 to 100 ng/mL) for 24 h. Cell viability was determined by a colorimetric assay after MTT staining. GI50 values were derived from the dose-response curves and were used to group the cell lines into TRAIL-resistant (GI50 >500 nM) or TRAIL-sensitive (GI50 <100 nM). (B) LC3 protein expressions in cells grown under healthy conditions. Individual cell lines were cultured in nutrient-rich medium. At 80% confluence, cells were harvested and analyzed by western blotting. LC3 immunoblots detect two bands: LC3-I at an apparent mobility of 18 kDa and the lipidated form LC3-II at 16 kDa (moves faster than LC3-I on SDS-PAGE). LC3-II is known to associate with autophagosomes and therefore serves a marker of active autophagy. β-actin was detected as a loading control. (C) Densitometry analysis of the immunoblots in (B) yielded the relative levels of LC3-II to LC3-I in individual cell lines. Shown are means and standard deviations of three independent experiments. TRAIL-sensitive cells are characterized by a LC3-II/LC3-I ratio less than 1 arbitrary unit. Statistical difference in LC3-II/LC3-I ratios between TRAIL-resistant and TRAIL-sensitive cell lines was determined by Fisher's PLSD test.

TRAIL resistance correlates with an accumulation of autophagosomes in breast cancer cell lines. We determined the activity of TRAIL in inducing apoptosis in a panel of 11 breast cancer cell lines (Fig. 1A and Ref. [50]). The obtained 50% growth inhibition (GI50) values allowed us to separate the cell lines into TRAIL-resistant lines (GI50 > 500 nM; AU565, BT474, HCC1428, MCF-7 and MDA-MB-453) and TRAIL-sensitive lines (GI50 < 100 nM; MDA-MB-231, BT549, HCC38, HCC1954, MDA-MB-157 and Hs578T). To determine the basal levels of autophagosomes, individual cell lines were grown in nutrient-rich media per ATCC recommendations. At 80% confluence, cells were harvested and analyzed by three complementary approaches following the guidelines for the use of assays for monitoring autophagy [51]. We first performed immunoblot analysis for the microtubule associated protein 1 light chain 3 (LC3). LC3 protein is involved in the formation of autophagosomes and its turnover from a cytosolic form LC3-I to a lipidated form LC3-II (*i.e.* addition of phosphatidylethanolamine at Gly120 residue of LC3-I) has been widely used as a molecular marker of autophagosomes. The results showed distinct patterns of LC3 expression between TRAIL-resistant and TRAIL-sensitive cell lines (Fig. 1B & C). In TRAIL-sensitive cell lines, LC3 existed primarily in its cytosolic form, LC3-I. By contrast, TRAIL-resistant cell lines were characterized by an increased level of the lipidated form, LC3-II. To rule out the possibility of cell density effects, we cultured the cells at 20%, 50%, and 80% confluence and observed a similar pattern in LC3 expression of two representative cell lines (BT474 and MDA-MB-231) (*Supplement I*). Notably, the high ratios of LC3-II/LC3-I correlated with the observed TRAIL resistance in the cell lines examined.

**Figure 2:**
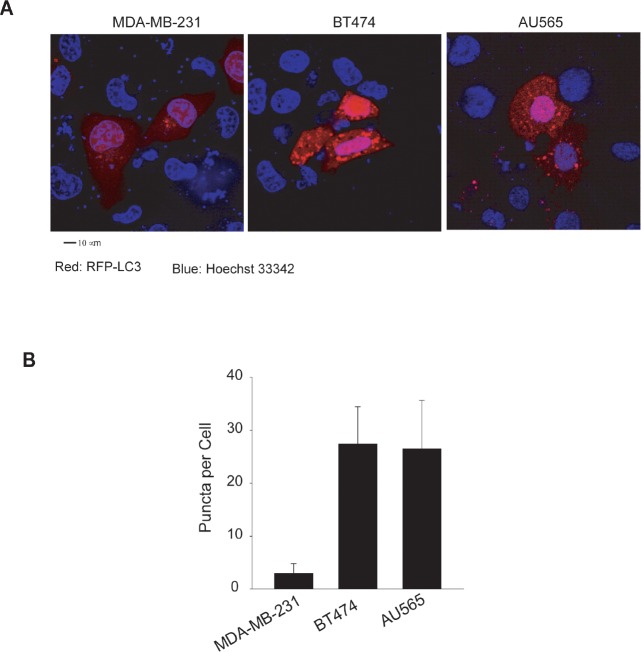
Transfected RFP-LC3 displays distinct patterns of localization between TRAIL-sensitive and TRAIL-resistant cell lines (A) Cells were transiently transfected with a plasmid encoding the red fluorescence protein and LC3 fusion protein (RFP-LC3). After 48 h post-transfection, cells were counterstained with Hoechst 33342 (blue). RFP-LC3 (red) shows a homogeneous staining in the cytoplasm of MDA-MB-231 cells, indicating the absence or low level of autophagosomes. Both BT474 and AU565 cells show punctate or dotted staining patterns of RFP-LC3 which is a typical marker of autophagosome structures. Scale bar, 10 μm. (B) Numbers of RFP-LC3 dots (Puncta) in the transfected cells as in A. Shown are the average numbers of puncta per cell estimated by examining at least ten images per cell line (mean ± SD).

**Figure 3:**
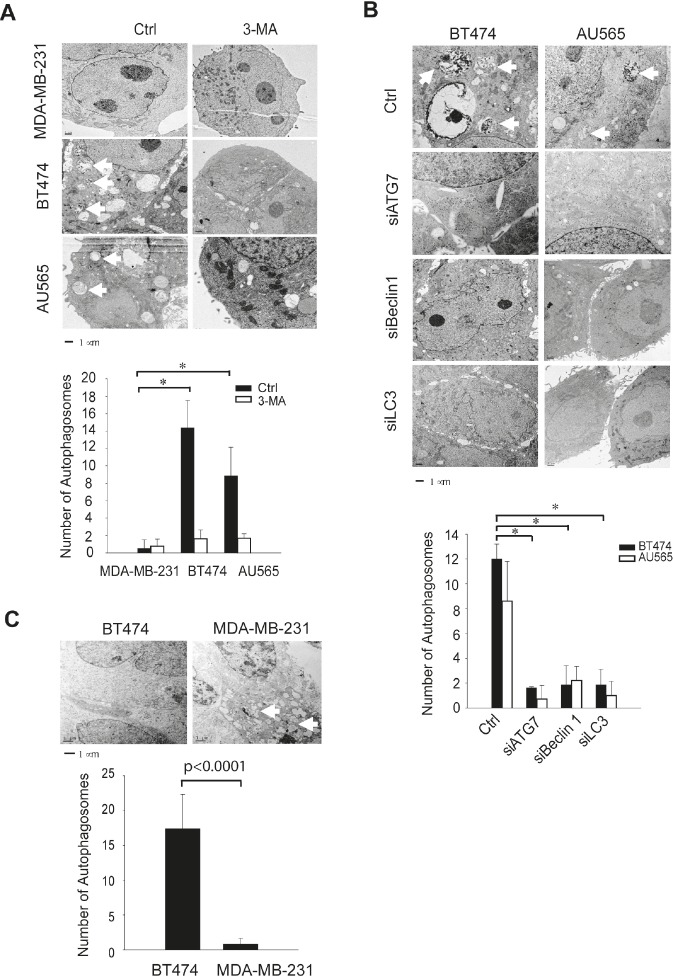
TEM images reveals autophagosome structures in TRAIL-resistant cell lines and xenograft tumor models (A) Electron microscopy (EM) images show the ultrastructural features of untreated cells or cells treated with 3-methyladenine (3-MA) at 10 mM for 24 h (Bar =1 μm). *Arrows* denote the autophagosome ultrastructures in cytoplasm. Lower panel shows the average number of autophagosome structures per view (371 μm^2^) obtained by examining at least 50 images per testing sample. *p<0.0001. (B) EM images of parental cells and cells transfected with siRNA specific to the autophagy regulatory genes ATG7, Beclin 1, and LC3, respectively. Images are representatives of at least 50 captures. *Lower panel* shows the average number of autophagosome structures as determined in (B) for individual samples. *p<0.0001. (C) Nude mice were injected s.c. with BT474 or MDA-MB-231 cells per the protocol described in the Materials and Methods. When tumors reached 0.6 cm*3* in size, tumor tissues were harvested and analyzed by EM imaging. Bar =1 μm. *Lower panel* shows the quantification of autophagosome numbers in the respective tissues.

We confirmed the high basal levels of autophagosomes in TRAIL-resistant cell lines using fluorescence microscopy and electron microscopy. Two representatives of TRAIL-resistant cell lines (BT474 and AU565) were compared to a TRAIL-sensitive cell line (MDA-MB-231). To facilitate fluorescence microscopy, cells were transiently transfected with a plasmid that expresses RFP-LC3 fusion protein. Compared to MDA-MB-231 cells, which showed an evenly distributed staining of RFP-LC3 red fluorescence, both BT474 and AU565 cells exhibited punctuate structures that are typical features of autophagosomes (Fig. 2). Further, electron microscopy images clearly showed the presence of a large number of autophagosomes in BT474 and AU565 cells but not in MDA-MB-231 cells (Fig. 3A). The number of autophagosomes decreased to baseline levels when the cells were treated with 3-methyladenine (3-MA), a pharmacological inhibitor of autophagy. Consistently, silencing of key autophagy regulatory genes (*ATG7*, *LC3*, or *Beclin1*) effectively abrogated the accumulation of autophagosomes in BT474 and AU565 cells (Fig. 3B). These data demonstrate that there are upregulated levels of basal autophagy in certain breast cancer cell lines even under healthy growing conditions, which correlates with the observed resistance to TRAIL-induced apoptosis. To assess the relevance of the high basal autophagy in a tumor setting, we established mouse xenograft models using BT474 and MDA-MB-231 cells. When tumor reached similar sizes (~0.6 cm^3^), tumor tissues were harvested and analyzed by electron microscopy. Consistent with the cell line data, a large number of autophagosomes were visualized in the tumors derived from BT474 cells but were virtually undetected in MDA-MB-231 xenografts (Fig. 3C).

**Figure 4:**
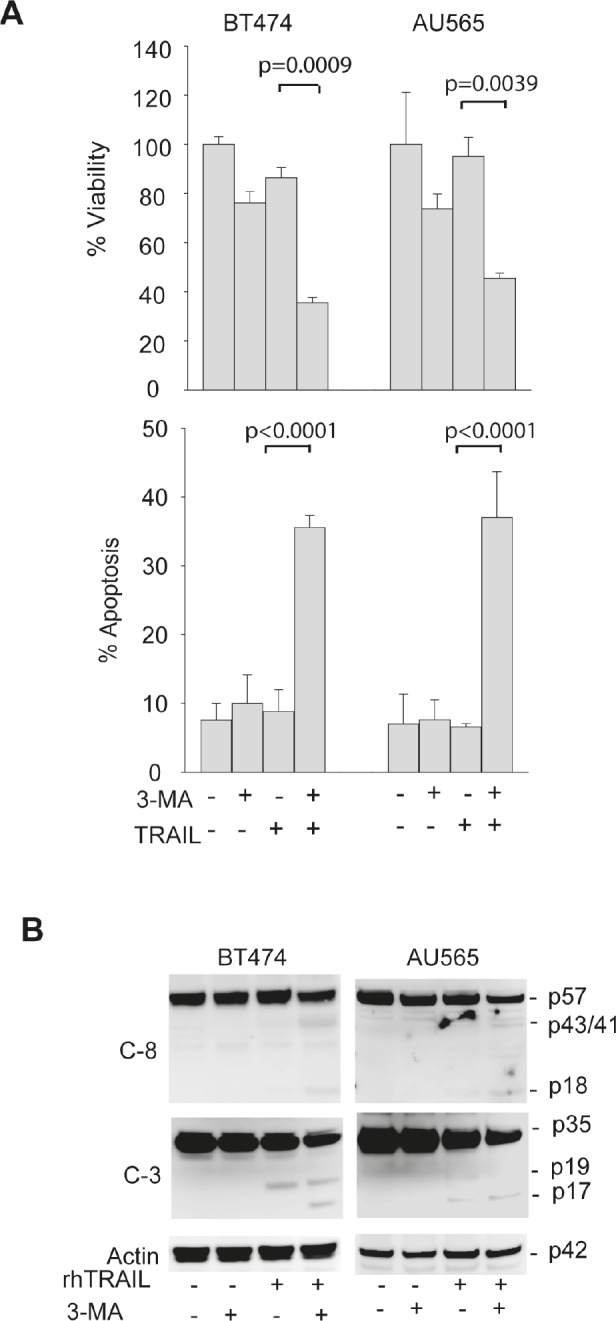
Pharmacological inhibition of basal autophagy sensitizes TRAIL-resistant cells to TRAIL induced apoptosis (A) *Top panel*, Cells were pretreated with 3-MA (10 mM) for 24 h followed by rhTRAIL (100 ng/ml) for an additional 24 h. Cell viability was expressed relative to the untreated cells under the same culture conditions. *Lower panel*, apoptosis was determined by flow cytometry after staining with propidium iodide (PI) and Annexin V-FITC. Shown are the percent of apoptotic cells stained positive for Annexin V-FITC or PI or both. (B) Western blots of caspase 8 (C-8) and caspase 3 (C-3) in samples as prepared in A. The activation of caspases is indicated by a decrease in the levels of intact pro-enzymes and the appearance of cleaved products (C-8: p43/41 and p18; C-3: p19/17). β-actin was used as a loading control.

**Figure 5:**
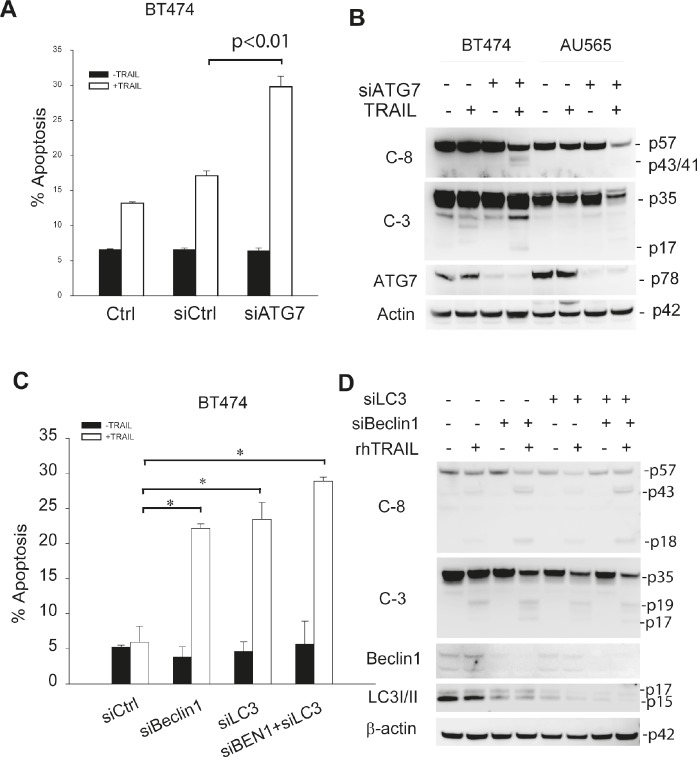
Enhancement of TRAIL sensitivity by silencing autophagy regulatory genes (A & B) The indicated cell lines were transiently transfected with a scramble siRNA (siCtrl) as a negative control or siRNA against *ATG7* for 72 h, followed by incubation with rhTRAIL (100 ng/ml) for an additional 24 h. The resultant cells were analyzed by flow cytometry for apoptosis (A) or immunoblotting for caspase cleavage (B). (C & D) BT474 cells were transiently transfected with a control siRNA (siCtrl) or siRNA specific to Beclin 1, LC3 or in combination. After 48 h post-transfection, cells were analyzed for apoptosis and caspase activation. *p<0.0001.

Suppression of autophagosome formation sensitizes TRAIL-resistant cells to TRAIL induced apoptosis. To determine whether the basal autophagy is actively involved in the development of TRAIL resistance, we tested the effects of autophagy inhibition on TRAIL induced cytotoxicity. First, TRAIL-resistant cell lines AU565 and BT474 were pretreated with 3-MA followed by TRAIL treatment. While blocking autophagosome formation (Fig. 3A), 3-MA treatment also induced a marked decrease in cell viability in both cell lines in response to TRAIL (Fig. 4A). The reduction in cell viability directly correlated with an increased apoptosis index and appearance of cleaved caspase-8 and caspase-3 (Fig. 4B). To rule out the off-target effects of 3-MA, we further tested the effects of silencing the key autophagy regulatory genes, including *ATG7*, *Beclin 1* and *LC3*. As shown in Fig. 3B, transfection of siRNA against the individual autophagic genes abolished autophagosome formation in TRAIL-resistant cells. Under similar conditions, knockdown of *ATG7* increased TRAIL-induced apoptosis (Fig. 5A) and caspase activation (Fig. 5B). Similar results were obtained by silencing *Beclin 1* or *LC3* (Fig. 5C & D). Collectively, these data demonstrate that basal autophagy is actively involved in the development of inherent resistance of breast cancer cells to TRAIL induced apoptosis.

**Figure 6:**
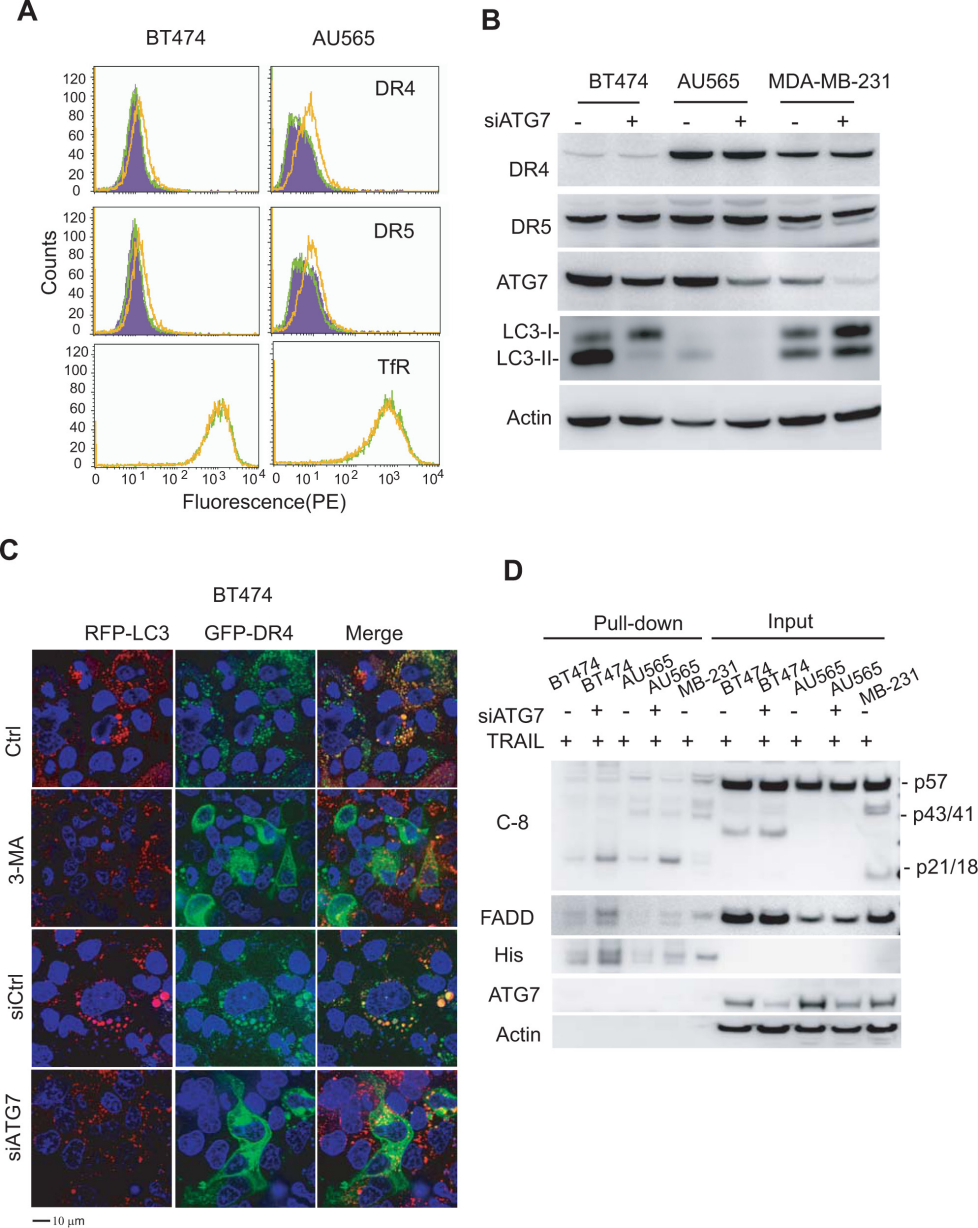
Inhibition of basal autophagy upregulates surface expression of death receptors (DRs) 4 and 5 (A) Flow cytometry analysis of death receptor expressions on cell surface. Cells were transiently transfected with a control siRNA (siCtrl) or siATG7. After 72 h post transfection, cells were incubated with PE-conjugated antibodies to DR4 (IgG1) or DR5 (IgG2b) or corresponding control IgG isotypes. Shown are representative histograms of siCtrl cells stained with control PE-IgG1 or PE-IgG2b (purple), PE-anti-DR4 or PE-anti-DR5 (green), and siATG7 transfected cells stained with PE-anti-DR4 or PE-anti-DR5 (yellow). The right-shift of a histogram peak indicates the increase in surface expression of the receptors. (B) Western blots of DR4 and DR5 total proteins. Knockdown of ATG7 is indicated by the decrease in ATG7 protein and the simultaneous loss of LC3-II. (C) Confocal images show the redistribution of DR4 from cytosol to plasma membrane upon inhibition of autophagy. Stable BT474/RFP-LC3 cells were left untreated or treated with 3-MA (10 mM) for 24 h. Alternatively, cells were transiently transfected with siCtrl or siATG7 for 48 h. Resultant cells were transfected with plasmids for GFP-DR4 or GFP-DR5 for 18 h. (D) Functional DISC formation assay. The indicated cells were transiently transfected with siATG7 for 72 h and incubated with (His)_6_-TRAIL (1 μg/ml) for 1 h. Affinity isolated DISC complexes were analyzed by western blotting. As a positive control, MDA-MB-231 cells (expressing both DR4 and DR5 on cell surface and are sensitive to TRAIL) recruited adaptor protein FADD and pro-caspase-8 into the DISC complexes upon TRAIL treatment. Only little DISC components were detected in parental BT474 and AU565 cells which are deficient in surface DR4/DR5 (Fig. 5A). Knockdown of ATG7 enhanced FADD and caspase 8, particularly the cleaved forms p43/41 and p21/18, in the DISC complexes, while had no effect on their total protein expressions.

Autophagosomes mediate downregulation of surface expression of TRAIL death receptors. We have previously shown that deficiency of DR4 and DR5 on the cell surface is a critical mechanism of TRAIL resistance [12, 13, 29]. In AU565 and BT474 cells, both DR4 and DR5 were found to be absent on surface membrane despite the high expression levels of their total proteins [29]. To understand the molecular basis of TRAIL sensitization by inhibition of autophagy, we examined the surface expression of DR4 and DR5 by flow cytometry using PE-conjugated antibodies specific to each receptor. Strikingly, knockdown of ATG7 induced a slight but significant increase in the surface expression of both death receptors (Fig. 6A), whereas had little or no effect on their total protein levels (Fig. 6B). As a control, the surface expression of transferrin receptor (TfR) was not affected in response to ATG7 depletion. Immunoblotting analysis also showed that inhibition of autophagy had no affect on the expressions of several major regulatory proteins of TRAIL apoptosis signaling pathway, including c-FLIP, Bcl-2 and IAP family proteins (*Supplement II*). While these downstream regulatory components remain intact and unaffected, the restoration of surface expression of DR4 and DR5 is likely a direct cause of the enhanced sensitivity to TRAIL following inhibition of autophagy. The above observations allowed us to propose that DR4 and DR5 may be trapped in autophagosomes in the TRAIL-resistant cells and that, upon inhibition of autophagy, they may be translocated onto the plasma membrane where they become accessible to ligand binding. To test this possibility, we established a stable BT474 cell line expressing a RFP-LC3 red fluorescence fusion protein (BT474/RFP-LC3). The stable cells were subsequently transfected with a plasmid encoding GFP-DR4 fusion protein. Confocal microscopy analysis revealed co-localization of RFP-LC-3 and GFP-DR4 in punctuate structures (yellow color) in BT474 cells (Fig. 6C). A similar result was obtained for GFP-DR5 and RFP-LC3 (data not shown). Inhibition of autophagy by 3-MA or by silencing ATG7 disrupted the co-localization pattern and, strikingly, the resultant cells displayed a strong staining of GFP-DR4 green fluorescence on the plasma membrane. Further, we determined the effect of silencing ATG7 on the ability of TRAIL in inducing assembly of death inducing signaling complexes (DISC), which is essential for transmitting an apoptosis signal. To this end, BT474 and AU565 cells were transfected with siATG7 and treated with (His)_6_-TRAIL. DISC components were affinity purified and analyzed by immunoblotting. In parental cells, TRAIL failed to efficiently assemble a DISC as shown by little or barely detectable signals of the adaptor molecule FADD and pro-caspase 8 (Fig. 6D). Knockdown of ATG7 significantly increased the levels of FADD and caspase 8 fragments (p43/41 and p21/18) in the DISC complexes. Collectively, these data support a notion that basal autophagy is actively involved in the downregulation of DR4 and DR5 on cell surface thereby making the cells resistant to TRAIL induced apoptosis.

**Figure 7:**
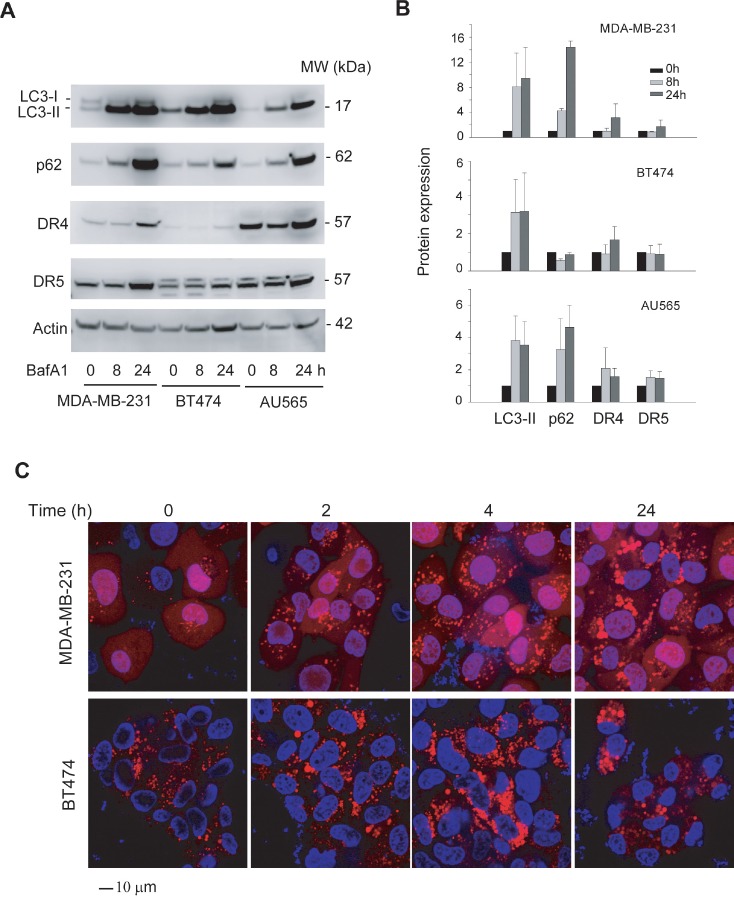
Inhibition of lysosomal activity induces differentially accumulation of autophagosome in MDA-MB-231 and TRAIL-resistant cell lines (A) Cells were treated with bafilomycin A1 (BafA1) at 100 nM for 8 or 24 h, and the resultant whole cell extracts were analyzed by immunoblotting for LC3, p62, DR4 and DR5. Actin was used as a loading control. (B) Relative protein levels were estimated by densitometry analysis of the blots in A, and normalized to the corresponding actin intensity. The level of each protein in the untreated cells (time 0) was arbitrarily set as 1. Shown are representatives of two independent experiments. (C) MDA-MB-231/RFP-LC3 cells and BT474/RFP-LC3, both stably express RFP-LC3 protein, were treated with BafA1 (100nM) for the indicated times, countered stained with Hoechst 33342 (blue), and analyzed by confocal microscopy. Scale bar, 10 μm. MDA-MB-231 cells accumulated punctate structures at a much higher rate compared to BT474 cells.

Inhibition of lysosomal activity induces an accumulation of autophagosomes and a loss of surface DR4 and DR5 in MDA-MB-231 cells. As autophagy is a highly dynamic process that involves multiple steps, it is therefore possible that the accumulation of autophagosomes could result from a genetic background with upregulated induction of autophagy or, alternatively, reflect a block in the later stages of the process, such as impaired autophagosome fusion and lysosomal degradation [50]. To address this issue, we examined autophagic flux in these cell lines using lysosomal protease inhibitors (bafilomycin or chloroquine). As expected, lysosomal inhibition induced a time-dependent accumulation of LC3-II protein in all three cell lines (Fig. 7A & B). Surprisingly, the rate of LC3-II accumulation is much higher in MDA-MB-231 cells compared to BT474 and AU565 cells. A similar accumulation pattern was also observed for endogenous p62, DR4, and DR5 proteins. Using stable cell lines that express fluorescent RFP-LC3, we further showed that MDA-MB-231 cells rapidly accumulated punctuate structures upon bafilomycin treatment (Fig. 7C). These data support a low level of autophagic flux activity in BT474 and AU565 cells, although they both contain high degrees of autophagosomes. This observation is suggestive of an insufficient fusion and autolysosome formation or lack of lysosomal activity or both. MDA-MB-231 cells appear to undergo a rapid lysosomal turnover, which may explain the lack of basal autophagosomes. However, these results do not rule out the possibility of differences in the regulatory proteins upstream of autophagosome formation between TRAIL-resistant and TRAIL-sensitive cells. Additional studies are required to determine the molecular basis of autophagosome accumulation in TRAIL-resistant cancer cells.

**Figure 8:**
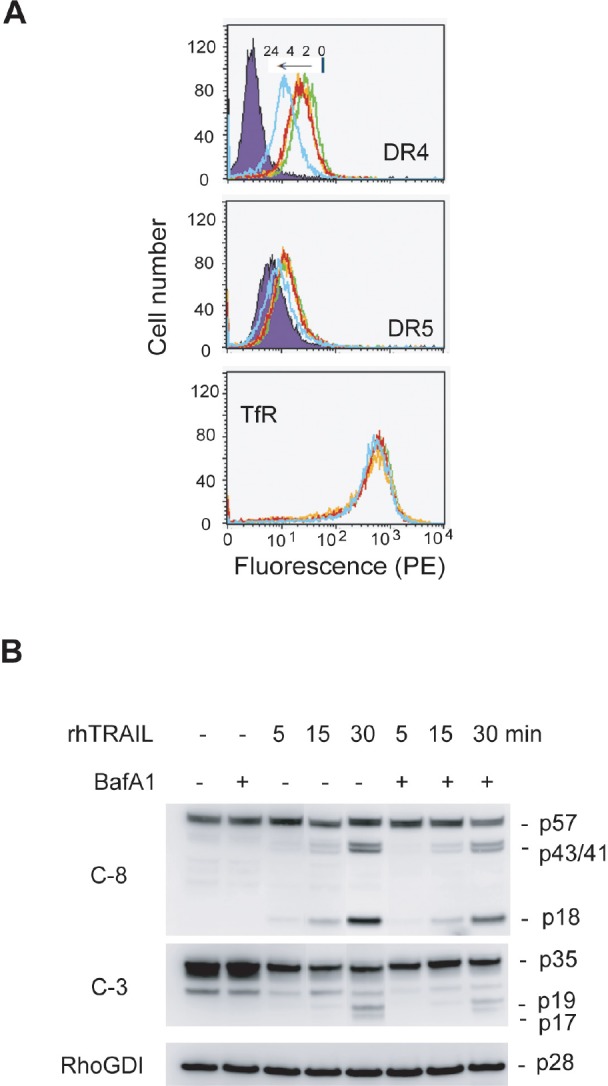
Inhibition of lysosomal activity downregulates DR4 and DR5 from the surface of MDA-MB-231 cells (A) Cells were treated with BafA1 (100 nM) at 37°C for 0, 2, 4 or 24 h, stained with PE-conjugated antibodies specific to DR4, DR5 or transferrin receptor (TfR), and analyzed by flow cytometry as in Fig. 6. Shown are representatives from three independent measurements. (B) Cells were pretreated with BafA1 (100 nM) for 24 h and followed by rhTRAIL (20 ng/mL) for additional 5, 15 or 30 min. A delay in cleavage of caspase 8 (C-8) and C-3) was detected in BafA-1 treated cells. RhoGDI, a caspase-resistant protein, was used as a loading control.

We further determined DR4/DR5 expression in MDA-MB-231 cells in response to lysosomal inhibition. Strikingly, both receptors were downregulated from surface membrane in a time-dependent fashion (Fig. 8A). The resultant cells became less sensitive to TRAIL-induced apoptosis, as indicated by a delay in the cleavage of caspase 8 and 3 immediately after TRAIL treatment (Fig. 8B). We wished to determine a decrease in apoptosis index after bafilomycin treatment, but this effort was hindered by the cytotoxicity of inhibitor itself. Collectively, both inherently occurring and induced autophagosomes appear to negatively regulate the surface expression of TRAIL death receptors.

## DISCUSSION

**Figure 9:**
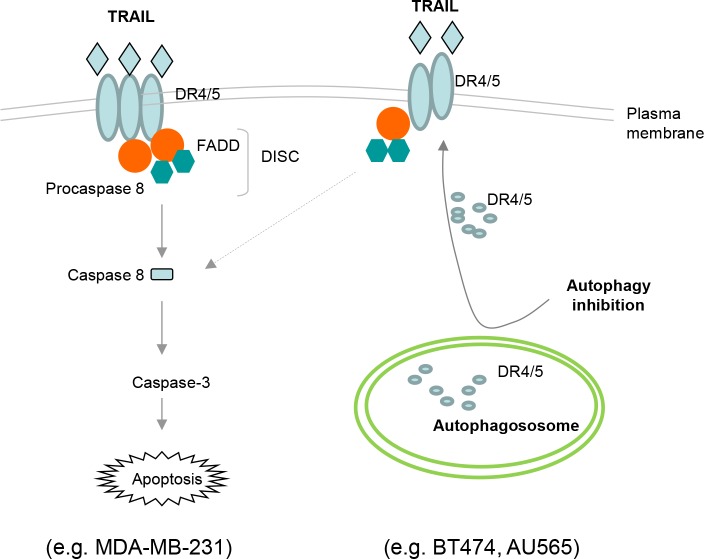
Schematic representation of the basal autophagosome mediated cellular resistance to TRAIL induced apoptosis In MDA-MB-231 cells, TRAIL binds DR4 and/or DR5 expressed on cell surface, thereby recruiting adaptor protein Fas-associated death domain (FADD) and pro-caspase 8 into a death inducing signaling complex (DISC). Within the DISC, caspase 8 undergoes self-cleavage and activation which triggers the caspase cascade, cleavage of structural proteins, and eventually apoptosis. Both BT474 and AU565 cells are characterized by high basal level of autophagosomes that sequester DR4 and DR5, which may contribute to their deficiency on cell surface. Disruption of autophagosome structures (e.g. by 3-MA or siATG7) restores the surface expression of DR4 and DR5 which make the cells susceptible to TRAIL induced apoptosis.

Overcoming tumor resistance is the key to success of development of TRAIL receptor targeted therapies for cancer treatment. Our laboratory investigates the mechanisms underlying TRAIL resistance with aims at identifying biomarkers for prediction of tumor resistance to the targeted therapies and also identifying novel molecular targets for therapeutic intervention for improved anticancer efficacy [12, 13, 29, 30, 50]. We have previously shown an aberrant expression of TRAIL death receptors in certain cancer cell lines in which these receptors are mainly localized in intracellular compartments such as nucleus and others yet to be characterized [29, 30]. Here we report a novel role of autophagy in regulation of subcellular localization of TRAIL receptors. Using complementary approaches, we show an upregulated formation of autophagosomes in a panel of TRAIL-resistant breast cancer cell lines and relevant animal models. We also provide evidence that the presence of basal autophagosomes is actively involved in the regulation of surface expression of TRAIL death receptors. Pharmacological inhibition of autophagosomes effectively restored the surface expression of DR4 and DR5 without altering their total protein levels. Importantly, blockage of basal autophagosome formation sensitized TRAIL-resistant cells (e.g. AU565 and BT474) to TRAIL induced apoptosis (Fig. 9). These findings highlight an important role of basal autophagosomes in the regulation of apoptotic response to TRAIL agonists. The molecular markers of autophagosomes (e.g. LC3-II/LC3-I ratio) could be evaluated as an indicator of inherent TRAIL resistance in tumor cells, and blockade of the basal autophagosomes may have a potential to improve the clinical efficacy of TRAIL receptor targeted therapies.

Increasing evidence shows that cancer cells can lose surface expression of TRAIL death receptors through a mechanism that is postulated to involve an accelerated DR4/DR5 endocytosis [13, 30] or insufficient receptor trafficking [31]. For example, DR5 is localized in intracellular compartments in NSCLC cells [52] and breast cancer cells [13, 29]. The present data demonstrate that TRAIL-resistant breast cancer cells (e.g. AU565 and BT474) contain high basal levels of autophagosomes (Figs. 1, 2 and 3). Strikingly, DR4 and DR5 are found to co-localize with LC3-II within autophagosomes and, upon disruption of autophagosome formation, translocate to the plasma membrane (Fig. 6 and not shown for DR5). This intriguing observation may be explained by recruitment of the endocytosed receptors or by an alternative mechanism in which autophagosomes engulf plasma membranes containing the receptors. In support the latter possibility, it has been shown that autophagosomes acquire membranes from sources including the plasma membrane [53].

In a canonical mode, autophagosomes fuse with lysosomes to form autolysosomes wherein degrade autophagic cargos for recycling of nutrients (to meet metabolic demands). Surprisingly, the steady-state protein levels of DR4 and DR5 in AU565 and BT474 cells are comparable to those of MDA-MB-231 cells lacking basal autophagosomes (Fig. 6B). Moreover, the total protein levels of DR4 and DR5 was virtually unchanged by silencing *ATG7* (data not shown) or treatment with lysosomal inhibitors (Fig. 7). Interestingly, inhibition of lysosomal activity resulted in an accumulation of DR4 and DR5 proteins in MDA-MB-231 cells at a much faster rate when compared to BT474 and AU565 cell lines. This phenomenon is elusive at this time and we speculate that there might be an impaired autolysosome formation or a deficiency in lysosomal activity or both. In this aspect, accumulation of autophagosomes has been shown to be related to inefficient fusion of autophagosomes with endosomes and/or lysosomes [54], or alternatively, to inefficient degradation of the cargo once fusion has occurred [55]. It is also possible that some cancer cells (e.g. BT474 and AU565) harbor a genetic background in favor of the induction of autophagosomes. Autophagosomes are thought to be efficient carriers of a broad spectrum of cellular antigens which may include tumor cell surface receptors [56]. Although the composition of autophagosomes is not clearly defined, a tumor-derived autophagosome vaccine has shown potent antitumor activity in preclinical studies [57]. Our data suggest that death receptors may be sequestered in the basal autophagosomes of tumor cells and may present as immunogens in the autophagosome-enriched vaccine. Studies are underway in our laboratory to isolate and characterize basal autophagosomes for their protein contents. Nonetheless, the accumulation of basal autophagosomes closely correlates with the deficiency of surface DR4 and DR5 and cellular resistance to rhTRAIL and monoclonal antibodies to DR4 or DR5 (Fig. 1).

Despite the opposing roles of autophagy in regulating cell death or cell survival, abundant evidence favor its cytoprotective role in the context of chemotherapy or radiation therapy. Recent research has shown that some cancer cells, particularly those driven by the K-Ras oncogene, depend on elevated levels of autophagy for survival and transformation even in the absence of external stressors [58]. Autophagy is also linked to primary resistance to HER2-targeted therapies in breast cancer treatment [59]. As a result, multiple clinical trials are ongoing to evaluate autophagy inhibitors (e.g. chloroquine and hydroxychloroquine) for cancer treatment [42]. TRAIL has been shown to induce autophagy in cells derived from colon [44, 45], glioma [46], bladder and prostate [47] and breast carcinoma [48, 49]. In support of a cytoprotective role for autophagy, we show that disruption of basal autophagosomes sensitized TRAIL-resistant cells (BT575 and AU565) to TRAIL-induced apoptosis. However, blockade of autophagic flux by inhibiting lysosomal activity made TRAIL-sensitive cells (MDA-MB-231) less sensitive to TRAIL (Fig. 7 & 8). This data suggests that targeting the different steps of the autophagy machinery may elicit opposite effects on TRAIL-induced apoptosis, depending possibly on cell types, tumor stages, and the status of basal autophagosomes. Additional studies are warranted to evaluate the therapeutic potential of autophagy inhibitors, especially inhibitors of autophagosome assembly *versus* those blocking lysosomal activity (e.g. bafilomycin and chloroquine).

The autophagosome mediated downregulation of surface TRAIL death receptors may also have an implication in understanding the role of autophagy in promoting tumor progression. TRAIL is primarily produced by immune cells (e.g. T cells and Natural Killer cells) and is present in circulation and tissue microenvironment where it serves as immunosurveillance for malignant cells by inducing cell death through the surface DR4 and/or DR5. When the death receptors are deficient in the cell surface, a “malignant” cell could escape from TRAIL mediated surveillance, which promotes tumor formation and metastasis. Consistent with our data, basal autophagy has also been found in pancreatic [60], melanoma [61], and non-small lung cancer cells [62]. Thus, it is reasonable to propose that accumulation of autophagosomes contributes to tumor progression through downregulation of death receptor mediated apoptosis.

In summary, we show an accumulation of autophagosomes in TRAIL-resistant breast cancer cells under nutrient-rich conditions. The presence of autophagosomes appears to negatively regulate the surface expression of TRAIL receptors, thereby blocking TRAIL induced apoptosis. It will require further study to elucidate the molecular basis of the upregulated basal autophagosomes and the signaling events involved in DR4/DR5 subcellular localization. Nonetheless, these findings have several potential therapeutic implications. Immunostaining of autophagosome markers such as LC3-II could be evaluated as predictive markers of tumor resistance to TRAIL related therapies. Furthermore, the data raise a caution on the selection of autophagy inhibitors for combination with TRAIL receptor targeted therapies.

## MATERIALS AND METHODS

### Cell lines and reagents

The human breast cancer cell lines AU565, BT474, HCC1428, MCF-7, MDA-MB-453, BT549, HCC38, HCC1954, MDA-MB-157, MDA-MB-231 and Hs578T were purchased from the American Type Culture Collection (ATCC), where the cell lines were tested and authenticated by growth rate, morphology, isoenzymology, short tandem repeat profiling, and Mycoplasma testing (www.ATCC.org). All the cell lines were cultured per ATCC recommendations and tested to be free of mycoplasma contamination at receiving and on a monthly basis. Recombinant human TRAIL (rhTRAIL) (R & D systems, 375-TEC) which contains 168 amino acids corresponding to the extracellular domain of human TRAIL (Val114–Gly281) was expressed by Escherichia coli and purified as a homotrimeric protein. 3-methyladenine (3-MA, M9281), a general inhibitor of autophagy, and anti-β-actin antibody (A2066) were purchased from Sigma-Aldrich. Antibodies against human caspase 3 (9662), caspase 8 (9746) and ATG7 (8558) were from Cell Signaling Technology. Anti-Beclin 1 antibody was from BD Biosciences (612112). Anti-LC3 was from Novus (NB100-2220). Anti-DR4 (IMG-275) and Anti-DR5 (IMG-120A) were from IMGENEX. Phycoerythrin (PE)-conjugated monoclonal antibodies to DR4 (FAB347P) and DR5 (FAB6311P) and the corresponding IgG1 (IC002) and IgG2b (IC0041P) controls were purchased from R&D Systems. Horseradish peroxidase–conjugated goat anti-rabbit IgG1 (sc-2054) or anti-mouse IgG1 (sc-2969) and anti-p62 (sc-28359) were from Santa Cruz Biotechnology. The synthetic small interference RNA (siRNA) oligos specific to LC3 (s39155), Beclin1 (s16537) or ATG7 (s20650) were purchased from Life Technologies, with the corresponding sequences: 5′-AAAUCCCGGUGAUAAUAGA-3′, 5′-CAGUUACAGAUGGAGCUAA-3′ and 5′-GGAACACUGUAUAACACCA-3′. pmRFP-LC3 plasmid for expression of a fusion protein of red fluorescence protein and LC3 (RFP-LC3) was a kind gift from Dr. David Gewirtz at Virginia Commonwealth University and was described previously. pEGFP-N1/DR4 plasmid encoding DR4-GFP protein (EGFP fused to the intracellular COOH terminus of DR4) was constructed in our laboratory.[13] pCMV6-AV-GFP encoding DR5-GFP (RG19808) (GFP fused to the intracellular COOH terminus of DR5) was purchased from Origene. Transfections of siRNA and plasmids were performed using Lipofectamine RNAiMAX and Lipofectamine 2000 from Life Technologies. Lysosomal inhibitors chloroquine (C6628) and bafilomycin A1 (B1793) were from Sigma.

### Cell viability

Cell viability was determined by a colorimetric assay using 3-(4,5-dimethylthiazol-2-yl)-2,5-diphenyltetrazolium bromide (MTT). Briefly, cells (10,000 cells/ per well) were seeded onto 96-well plates and pre-treated with 3-MA or individual siRNA followed by treatment with rhTRAIL (100 ng/ml). The medium was changed to 100 μL of MTT solution (Sigma, M2128; 2 mg/ml) and incubated at 37°C for 2 h. The resulting crystals were dissolved in 100 μL of DMSO and absorbance at 562 nm was used to calculate the cell viability relative to the untreated cells. Apoptosis Assay. Apoptosis was determined by flow cytometry as previously described [13]. Briefly, cells were grown on 6-well plates to ~50% confluence and treated similarly as described above. For siRNA assays, cells were transiently transfected with the specific siRNA for 72 h and then incubated with rhTRAIL (100 ng/ml) for an additional 24 h. The resultant cells were labeled by Annexin V-FITC and propidium iodide (PI) using the Apoptosis Dectection Kit (Calbiochem, PF032) and analyzed on FACS Calibur (BD Biosciences) with CellQuest software (Becton Dickinson).

### Immunoblotting

Whole cell lysates were prepared using a lysis buffer containing 10 mM Tris-HCl, pH 7.4, 150 mM NaCl, 0.25% deoxycholic acid, 1% NP-40, 1 mM EDTA. Protein concentrations were estimated using the bicinchoninic acid protein assay (Pierce Biotechnology, 23235). Equal amounts of cell lysates (60 μg per lane) were resolved by SDS-PAGE using a 4%-12% NuPAGE Bis-Tris gel (Life Technologies, NP0321BOX) and transferred to PVDF membranes. Immunoblotting analyses were performed using primary antibodies at an appropriate dilution (1:500 to 1:1,000). When necessary, the membranes were stripped by Restore Western Blot Stripping Buffer (Pierce Biotechnology, 21059) and reprobed with appropriate antibodies. Immunocomplexes were visualized by chemiluminescence using Immobilon Western Chemiluminescent HRP Substrate (Millipore, WBKLS0500). Densitometry analysis was performed with the LAS-4000 Luminescent Image Analyzer (Fujifilm). Cell surface expression of death receptors. The cell surface expression of DR4 and DR5 was assessed by flow cytometry using the phycoerythrin (PE)-conjugated antibodies as described previously.[13] Briefly, cells (~5 × 10^6^) were incubated in a blocking solution (5 % normal goat serum and 1% bovine serum albumin in PBS) for 20min on ice, and then incubated with 10 μL of PE-conjugated primary antibodies for 45 min at 4°C on ice in dark. Duplicate samples were incubated with the respective IgG1-PE or IgG2b-PE as negative controls. The cells were washed twice with PBS and resuspended in 200μL PBS for FACS analysis.

### DISC formation assay

TRAIL induced DISC formation was analyzed as described previously [31]. Briefly, cells (1 × 10^7^) were incubated with 1 μg/mL of (His)_6_-rhTRAIL at 37°C for 1 h. The cells were washed twice with ice-cold 1xPBS and lysed for 30 min on ice in a lysis buffer [30 mmol/L Tris (pH 7.5), 150 mmol/L NaCl, 10% glycerol, 1% Triton X-100] supplemented with protease inhibitors and 5 mmol/L imidazole (Sigma, I2399). The cells lysates were cleared twice by centrifugation at 4°C. Equal amounts of extracts were incubated with 60 μL Nickel-NTA agarose beads (Qiagen, 1018244) at 4°C for 1 h. The affinity complexes were subsequently washed three times with lysis buffer including protease inhibitors and incubated with 1 mol/L imidazole for 10 min at room temperature. After centrifugation, supernatant was subject to western blotting analysis.

### Immunofluorescence microscopy

Stable BT474/RFP-LC3 cells were cultured onto Nunc Labtek Chambered cover glass (Thermo Fisher Scientific, 155383) and transiently transfected with GFP-DR4 or GFP-DR5 plasmids. After 24 h post-transfection, cells were counterstained with Hoechst 33342 (Sigma, H3570) and subject to confocal imaging using the Zeiss Cell Observer Spinning Disk Confoca Microscope lsystem. An oil immersion 63x objective lens (NA=1.4) was used for all optical imaging. Three laser lines (405, 488 and 561 nm) were used for Hoechst 33342, GFP and RFP and all samples were imaged with these 3 channels. The emission filters for image acquisition of Hoechst 33342, GFP and RFP are 450/50 nm, 525/50 nm and 629/62 nm, respectively. All images were saved and stored as zvi format for further data analysis. All images were representative of 3 independent experiments.

### Electron Microscopy

Cells were grown on thermonax plastic coverslips (Thermo Fisher Scientific, 174950) and fixed in a mixture of paraformaldehyde (2.5%, Electron Microscopy Sciences, 15710) and gluteraldehyde (2.0%, Electron Microscopy Sciences, 16000) in phosphate saline (PBS) for 1 h. After extensive wash in PBS, the slides were rinsed in 0.1 M sodium cacodylate buffer (SB; pH= 7.4, Electron Microscopy Sciences, 11652) and post-fixed in 1.0% osmium tetroxide (Electron Microscopy Sciences, 19150) mixed with 0.8% potassium ferricyanide (Electron Microscopy Sciences, 20150) for 1 h. The resultant samples were dehydrated in a series of ethanol (30%, 50%, 75%, 95% for 5 min and 100% for 30 min with three repeats) and infiltrated with Epon-Aradite (Electron Microscopy Sciences, 13940) for 1-2 days (30% of Epon-Araldite and ethanol for 2 h, 50% for 4 h, 75% for overnight and 100% for 24 h with 2 changes). The samples were polymerized at 60°C for 24h and were cut into ultrathin sections (~ 80 nm) using Leica EM UC6 Ultramicrotome (Leica, Buffalo Grove, IL) and collected on copper slot grids. Sections were counterstained with uranyl acetate(Electron Microscopy Sciences, 22400) and lead citrate(Electron Microscopy Sciences,17800), and examined under the FEI Tecnai12 transmission electron microscope (FEI, Hillsboro, Oregon) operating at the beam energy of 120keV. Images were acquired using the Gatan 2k × 2k cooled CCD camera (Gatan, Warrendale, PA).

### Xenograft mouse tumor model

Animal studies were performed following the institutional approved animal protocol (Center for Biological Evaluation and Research, WO-2006-50). Briefly, BT474 (1×10^7^ cells in 150 μL medium) or MDA-MB-231 cells (5×10^6^ cells in 150 μL medium) were injected subcutaneously (s.c.) at the right flank of nude mice (Athymic NCr-nu, female, 4-weeks old). Ten mice were used for each cell line. Given that BT474 cells require estrogen to grow *in vivo* (Liang Y et al. 2007), mice were implanted a 60-day release pellet of 17β-estradiol (Innovative Research Company, SE-121) under the skin of lateral side of the neck using trochar (Innovative Research Company, MP-182) 2 days prior to injection of BT474 cells. Tumor growth was monitored daily until the tumor volume reached 0.6 cm^3^. Tumors were then removed and fixed in formalin and analyzed by electron microscopy.

### Statistical analysis

Statistical analyses were performed with Statview software. Statistical difference was determined by a one-way analysis of variance (ANOVA) followed by Fisher's PLSD test. All cell-based assays were done in triplicate. Results were reported as mean +/− standard deviation (SD). Statistical significance was defined as *p* < 0.05.

## Supplementary Figures


