# Supplementary material for: Accumulation of autophagosomes in breast cancer cells induces TRAIL resistance through downregulation of surface expression of death receptors 4 and 5
Source: Oncotarget. 2013 Jul 27;4(9):1349–64. doi: 10.18632/oncotarget.1174 (PMC3824535; doi:10.18632/oncotarget.1174)

Supplement I LC3 expressions in cells grown at different confluency

The indicated cell lines were cultured in complete growth media to a confluency of 20%, 50%, and 80%, respectively. Cells were harvested and whole lysates were analyzed by western blotting. BT474 cells show a higher LC3-II/LC3-I expression than MDA-MB-231 cells. The LC3-II/LC3-I expression patterns were not affected by cell density.

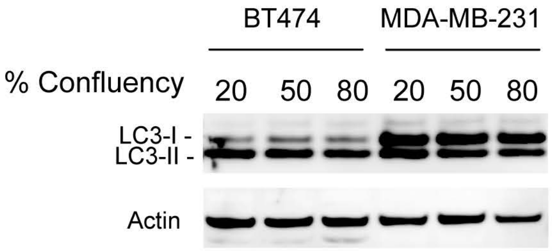

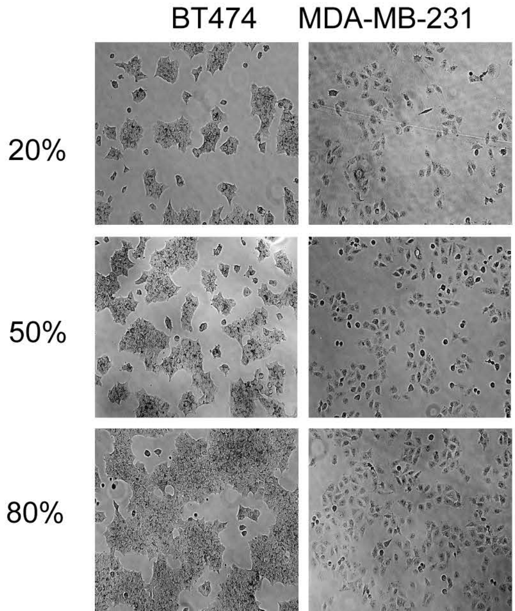

Supplement II Western blots of proteins related to TRAIL apoptosis signaling pathway

BT474 cells were left untreated or treated with 3-MA (10 mM) for 24 h and then incubated with rhTRAIL (100 ng/mL) for an additonal 24 h. Whole cell lysates were analyzed by western blotting using antibodies specific to individual proteins.

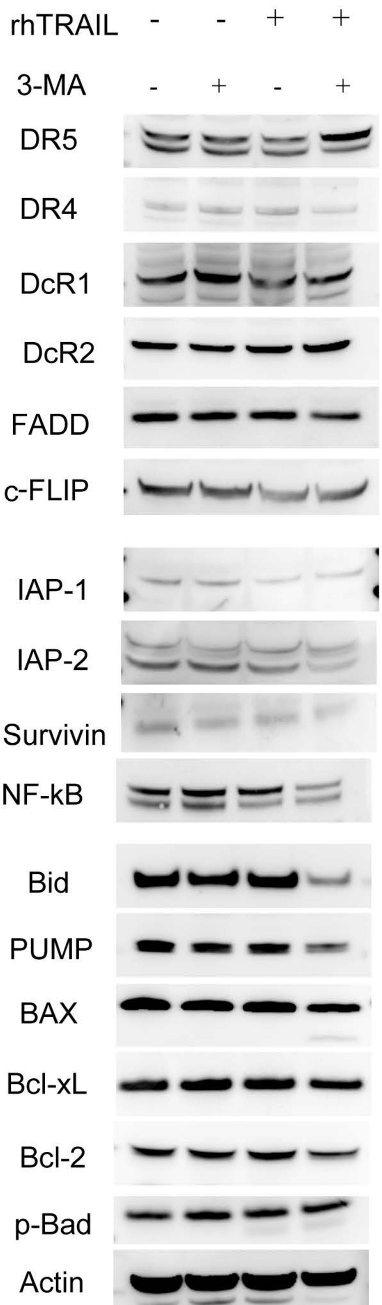

Supplement: Supplementary file 1 [file oncotarget-04-1349-s001.pdf]
